# An AI-driven evidence ecosystem for cardiometabolic nutrition: bridging the gap from multiomics to patient empowerment

**DOI:** 10.3389/fnut.2026.1924184

**Published:** 2026-09-28

**Authors:** Qingyong Zheng, Jianguo Xu, Tengfei Li, Ming Liu, Yongjia Zhou, Min Tian, Shaojuan Huo, Li Zhou, Jinhui Tian

**Affiliations:** 1 Evidence-Based Medicine Center, School of Basic Medical Science, Lanzhou University, Lanzhou, China; 2 Key Laboratory of Evidence-Based Medicine of Gansu Province, Lanzhou, China; 3 Duke-NUS Medical School, National University of Singapore, Singapore, Singapore; 4 Population Health Research Institute, Hamilton, Canada; 5 Chinese Academy of Medical Sciences & Peking Union Medical College, Beijing, China; 6 Lee Kong Chian School of Medicine, Nanyang Technological University, Singapore, Singapore; 7 Department of Pharmacy, Gansu Provincial Hospital, Lanzhou, China; 8 Department of Orthopedics, Lanzhou University Second Hospital, Lanzhou, China; 9 Department of Pediatric Gastroenterology, Gansu Province Maternity and Child Health Hospital, Gansu Province Central Hospital, Lanzhou, Gansu, China; 10 Research Unit of Evidence-Based Evaluation and Guidelines, Chinese Academy of Medical Sciences, Lanzhou, China

**Keywords:** artificial intelligence, cardiometabolic nutrition, evidence synthesis, patient empowerment, precision medicine, real-world data

## Abstract

Cardiometabolic diseases remain a primary global health challenge, with nutritional interventions serving as a critical pillar of management. However, the rapidly expanding volume of nutritional research, spanning high-dimensional multiomics, real-world continuous monitoring, and highly heterogeneous clinical trials, has created fragmented knowledge silos, leading to a profound translation lag between scientific discovery and clinical practice. To overcome this methodological bottleneck, we propose a conceptual framework for an “AI-Driven Evidence Ecosystem.” This perspective outlines a four-phase closed-loop architecture: (1) AI-assisted mechanistic decoding of multiomics and the development of digital gut twins; (2) data-optimized clinical research integrating traditional trials with real-world digital biomarkers; (3) a methodologically agnostic, multi-agent living evidence synthesis engine capable of automating complex data extraction and mitigating the translation gap; and (4) the deployment of conversational AI for public knowledge translation and personalized patient empowerment. By addressing concurrent ethical challenges, algorithmic biases, and the risk of researcher de-skilling, this ecosystem paradigm leverages artificial intelligence not merely as an automation tool, but as an indispensable bridge. Ultimately, it transforms isolated, stagnant data into a dynamic, translatable evidence network, reshaping the future of precision cardiometabolic care.

## Introduction

Cardiometabolic diseases (CMDs) remain a primary global health challenge, with nutritional interventions serving as a cornerstone for disease management. However, the current landscape of nutritional research presents a striking paradox: while the volume of publications has grown exponentially, researchers and clinicians are increasingly overwhelmed by a sea of fragmented literature. Valuable insights, ranging from high-throughput multiomics and gut microbiome profiles to diverse dietary patterns and observational cohorts, remain isolated in their respective subfields. Rather than forming a cohesive knowledge base, these research efforts often operate in silos, a fragmentation starkly visualized in the current literature network (Figure 1), struggling to cross-pollinate and inform broad clinical practices.

**FIGURE 1:**
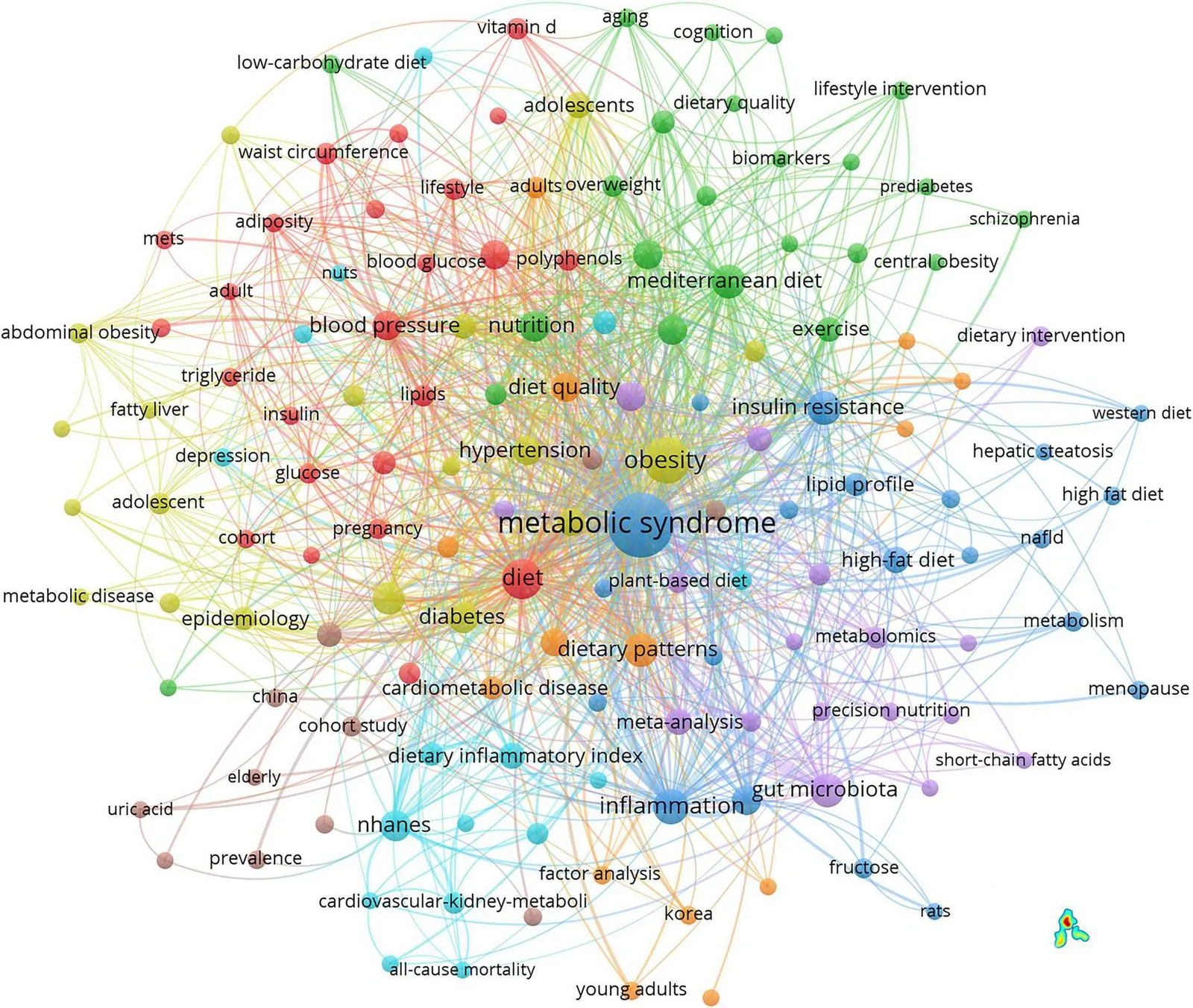
The landscape of evidence fragmentation in cardiometabolic nutrition. A keyword co-occurrence network illustrating the structural fragmentation of current research, based on literature spanning the past decade. The network visualizes highly polarized and topologically isolated sub-clusters: fundamental mechanistic domains (e.g., gut microbiota, metabolomics) are distinctly separated from population-level epidemiology and applied clinical research (e.g., observational cohorts, lifestyle interventions).

In an attempt to navigate this information overload, the scientific community frequently relies on systematic reviews and meta-analyses to distill high-certainty evidence. Yet, this approach is inherently limited. While effective at aggregating specific clinical endpoints, it leaves vast amounts of mechanistic, preclinical, and real-world data unintegrated. Furthermore, the manual labor required to screen, appraise, and extract scattered evidence imposes a severe cognitive burden on researchers. Consequently, this creates a profound translation gap. The labor-intensive nature of traditional evidence synthesis means that by the time comprehensive clinical guidelines are published, the foundational evidence may have already evolved, rendering the recommendations partially obsolete.

The rapid evolution of artificial intelligence (AI), particularly Large Language Models (LLMs), provides a timely opportunity to address this systemic fragmentation. Rather than merely serving as automated search engines or basic literature screening tools, advanced AI systems possess the capacity to process massive, unstructured datasets and identify latent connections across disparate biomedical domains. By acting as an intelligent bridge, AI can synthesize complex mechanistic pathways with diverse clinical outcomes, significantly alleviating the cognitive burden on individuals and fostering a more integrated research ecosystem.

Here, we outline a conceptual framework for an “AI-Driven Evidence Ecosystem” in cardiometabolic nutrition. Aligning with the journal’s focus on translating research “from mechanisms to clinical practice,” we envision a four-phase closed-loop approach (the core technical infrastructure of which is detailed in Table 1): (1) AI-assisted mechanistic decoding of high-dimensional data; (2) data-optimized clinical research generation; (3) intelligent evidence synthesis via multi-agent systems; and (4) the translation of guidelines into accessible public health knowledge (Figure 2). Through this framework, we aim to stimulate discussion on how the scientific community can harness AI to transform isolated research efforts into a cohesive, dynamic, and highly translational evidence network.

**TABLE 1:** Technical implementation and infrastructure of the AI-driven evidence ecosystem.

Ecosystem phase	Primary AI models & architectures	Data input requirements & standards	Key technical challenge	Proposed mitigation strategy
Phase 1: mechanistic decoding & multiomics	GNNs, deep autoencoders	High-dimensional omics data (FASTQ, VCF), metabolomics (mzML)	Extreme high dimensionality combined with low sample sizes	Employ transfer learning, robust dimensionality reduction, and integrate biological knowledge graphs.
Phase 2: clinical & real-world evidence	Time-series transformers, penalized regression models	CGM, EHRs formatted via FHIR	Data noise, high missingness rates, and asynchronous temporal data	Implement advanced imputation algorithms, robust signal processing, and federated learning.
Phase 3: living evidence synthesis engine	Multi-agent collaborative networks via LLMs, RAG	Unstructured scientific literature, clinical trial registries via API	Resolving conflicting evidence; AI “hallucinations” in data extraction	Institute strict expert adjudication (e.g., GRADE panels) and mandate RAG grounding.
Phase 4: public translation & empowerment	Fine-tuned conversational AI, reinforcement learning from human feedback	Personal dietary logs, OMOP, CDM	Outputs exceeding patient health literacy; unverified dietary advice	Adopt a “clinician-guided AI counseling” model; enforce strict readability constraint prompts.

AI, artificial intelligence; API, application programming interface; CDM, Common Data Model; CGM, continuous glucose monitoring; EHRs, electronic health records; FHIR, Fast Healthcare Interoperability Resources; GNNs, Graph Neural Networks; GRADE, Grading of Recommendations, Assessment, Development, and Evaluations; LLMs, Large Language Models; OMOP, Observational Medical Outcomes Partnership; RAG, Retrieval-Augmented Generation. (FASTQ, VCF, and mzML denote standard multiomics data file formats).

**FIGURE 2:**
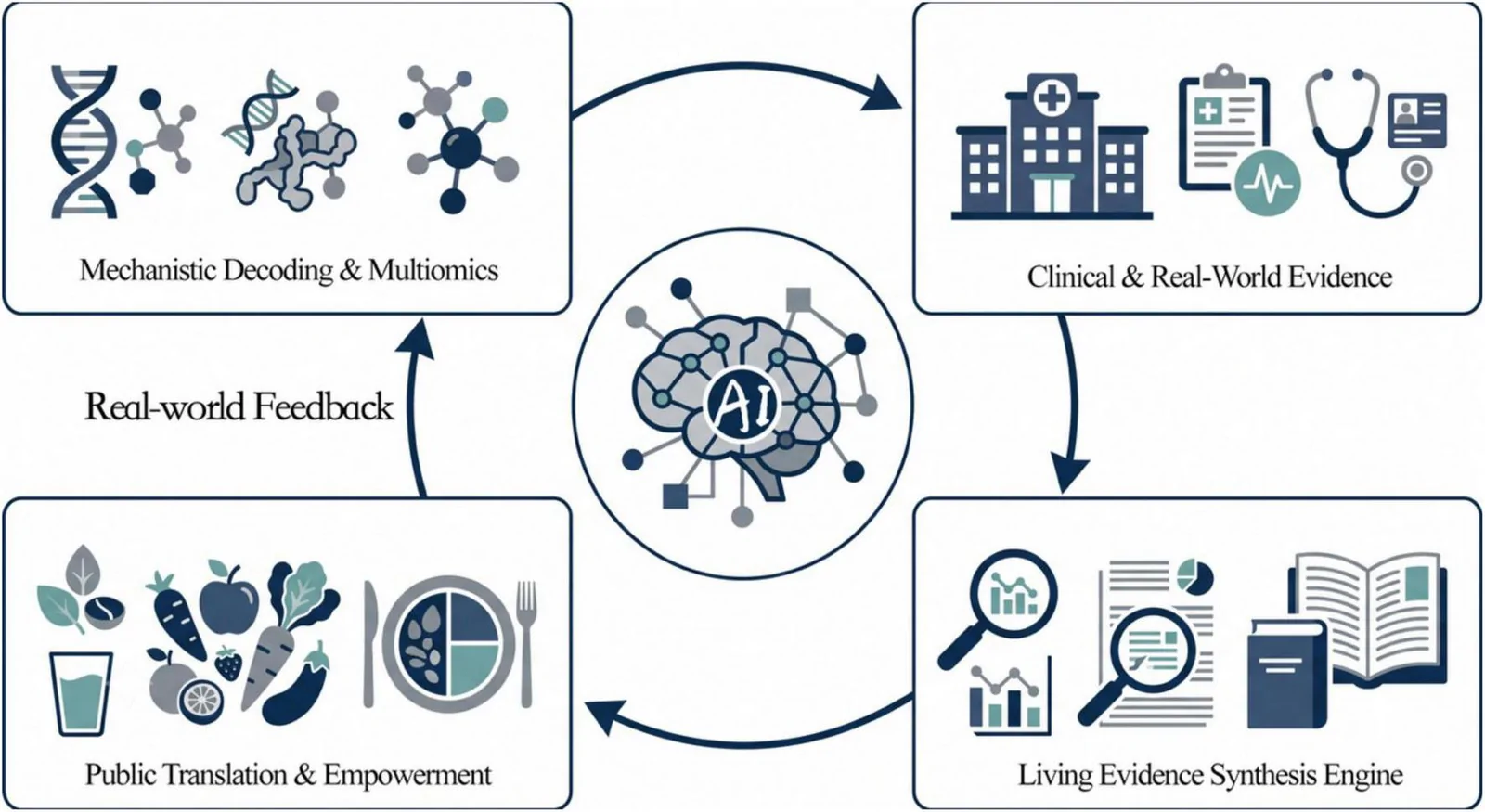
Conceptual framework of the AI-driven evidence ecosystem in cardiometabolic nutrition. A four-phase, closed-loop architecture designed to bridge the translational gap from fundamental research to patient care. The central artificial intelligence engine orchestrates continuous data flow across all domains: (1) Mechanistic Decoding & Multiomics, leveraging machine learning to integrate high-dimensional biological data; (2) Clinical & Real-World Evidence, optimizing trials and continuous wearable biomonitoring; (3) Living Evidence Synthesis Engine, utilizing multi-agent AI systems for automated, methodologically agnostic data extraction and integration; and (4) Public Translation & Empowerment, translating complex guidelines into accessible, precision dietary guidance via conversational AI.

## Phase 1: AI-driven decoding of multiomics and mechanistic pathways

The foundation of cardiometabolic nutrition lies in deciphering the highly complex, multidirectional interactions between dietary intake, host physiology, and the gut microbiome (1). Nutrition science has rapidly transitioned from evaluating isolated macro- and micronutrients to investigating high-dimensional multi-omics landscapes, encompassing genomics, transcriptomics, metabolomics, and metagenomics (2). However, interpreting these vast, heterogeneous datasets far exceeds human cognitive bandwidth and challenges the limits of traditional statistical analyses. Conventional linear models, while valuable for establishing baseline epidemiological associations, are frequently underpowered to capture the non-linear, synergistic, and highly individualized biological responses inherent in precision nutritional interventions (2).

Artificial intelligence, particularly advanced machine learning (ML) and deep learning algorithms, is uniquely equipped to decode these intricate biological networks. By employing techniques such as dimensionality reduction and feature selection on large-scale data matrices without relying on rigid *a priori* assumptions, AI can identify latent patterns and generate novel mechanistic hypotheses (3). For example, pioneering research utilizing machine learning has successfully integrated gut microbiome features, host genetic variants, and meal macronutrients to accurately predict highly individualized postprandial glycemic and lipemic responses (4). Similarly, penalized ML approaches applied to observational cohorts have uncovered distinct metabolite signatures linked to adverse dietary patterns, enabling the prediction of long-term cardiometabolic risk independent of traditional lifestyle factors (5).

Moving beyond static risk prediction, the field is rapidly advancing toward dynamic, AI-driven mechanistic modeling. The conceptualization of the “digital gut twin,” a continuously updated, AI-powered simulation integrating host multi-omics, microbiome profiles, and comprehensive nutrient databases, represents a frontier in precision cardiometabolic health and disease prevention (6). Operationally, these twins would be powered by multi-modal deep learning architectures. Specifically, Graph Neural Networks (GNNs), are utilized to map the topological relationships within microbial communities, while deep autoencoders facilitate the robust dimensionality reduction of paired multiomics data. However, a clear demarcation must be drawn between current capabilities and future aspirations. While static machine learning models for clinical risk prediction are empirically validated, the fully dynamic “digital gut twin” remains a theoretical construct. Translating these early *in silico* proof-of-concepts into deployable clinical tools will require extensive longitudinal, *in vivo* validation over the coming decade. These sophisticated computational models allow researchers to simulate the metabolic consequences of dietary modifications *in silico*, pinpointing specific microbiota-derived metabolites or inflammatory cytokines that mediate disease pathogenesis long before *in vivo* trials yield conclusive results (7). Ultimately, this AI-accelerated decoding phase transforms raw, high-throughput omics data into validated, actionable biological targets, laying a rigorous foundation for the subsequent design of precision clinical interventions.

## Phase 2: generating data-optimized clinical evidence and real-world insights

Transitioning from AI-decoded mechanistic targets to actionable clinical guidelines requires robust evidence generation at the population level. However, traditional nutritional randomized controlled trials (RCTs) frequently encounter substantial methodological hurdles, including poor long-term dietary adherence, imprecise population stratification, and the inherent limitations of “one-size-fits-all” dietary guidelines. Consequently, AI is driving a paradigm shift in clinical evidence generation through two primary, interconnected tracks: the optimization of prospective trial designs and the deep mining of real-world data (RWD).

In the context of prospective trial optimization, AI facilitates the transition toward true precision nutrition by characterizing distinct “nutri-phenotypes” prior to intervention. By analyzing baseline clinical features, dietary habits, and microbiome composition, machine learning algorithms can accurately predict highly personalized metabolic responses to specific dietary modifications (8). Recent rigorous clinical interventions have demonstrated that AI-driven, personalized postprandial-targeting diets yield more prominent improvements in glycemic control, hemoglobin A1c (HbA1c), and cardiometabolic markers than standard generalized approaches, such as the Mediterranean diet (8). By simulating interventions and stratifying participants *in silico* before trial execution, AI significantly enhances the signal-to-noise ratio and reproducibility of nutritional RCTs.

Concurrently, the rapid proliferation of digital health technologies has unlocked massive repositories of continuous, high-resolution RWD. The integration of digital biomarkers, derived from continuous glucose monitoring (CGM) devices, smartphone-based dietary logging, and wearable technologies, provides a dynamic view of real-world nutritional behaviors and metabolic fluctuations (9). AI models excel at analyzing these longitudinal, noisy datasets, transforming observational data into robust real-world evidence. Furthermore, AI is increasingly being leveraged in broader public health strategies, such as “Food is Medicine” (FIM) initiatives, to conduct automated nutritional assessments, generate personalized meal plans, and evaluate health outcomes from natural experiments in diverse populations (10). By bridging the gap between highly controlled RCTs and dynamic RWD, AI ensures that the clinical evidence generated is both scientifically rigorous and directly translatable to patient-centered clinical applications (9).

## Phase 3: building a living, methodologically agnostic evidence synthesis engine

Once primary clinical evidence and real-world data regarding dietary impacts are generated, the ecosystem must synthesize these disparate inputs to inform cardiometabolic nutrition guidelines. Traditionally, this synthesis stage represents a severe methodological bottleneck. Systematic reviews and meta-analyses in nutrition are notoriously resource-intensive due to the vast heterogeneity of dietary interventions and metabolic endpoints, often becoming outdated shortly after publication (11). Previous efforts to automate these workflows via traditional ML were largely constrained to single stages, such as abstract screening, and suffered from high error rates and limited adaptability across diverse medical topics (12, 13). The integration of LLMs and generative AI (GenAI) offers a timely opportunity to transition from these rigid automation prototypes to a professionally integrated, full-workflow “living” evidence synthesis engine (12, 14).

Crucially, this AI-powered engine is methodologically agnostic. It does not prioritize a single analytical design, such as pairwise or network meta-analyses; rather, it fundamentally re-engineers the infrastructure of evidence integration itself, making it scalable for scoping reviews, umbrella reviews, or qualitative syntheses (15). This overhaul is achieved by deploying a multi-agent collaborative network that digitally replicates an expert human review panel, where distinct LLM agents act as independent reviewers and arbiters. Recent empirical studies demonstrate that such agentic architectures significantly outperform traditional screening tools, achieving exceptional sensitivity and specificity in full-text screening and identifying complex inclusion/exclusion criteria (16). When addressing contradictory findings across diverse dietary trials, this engine employs a multi-agent debate protocol. Distinct LLM agents evaluate conflicting methodologies and effect sizes, ultimately flagging high-variance endpoints for mandatory human review. By integrating AI-driven confidence scoring with established human-in-the-loop frameworks like GRADE (Grading of Recommendations Assessment, Development, and Evaluation), the system ensures rigorous and transparent conflict resolution. Furthermore, these models excel at contextual comprehension, enabling them to assist humans in complex downstream tasks such as data extraction and quality assessment or risk-of-bias evaluation (17). Within our proposed ecosystem, this advanced architecture is perfectly suited to handle the vast heterogeneity of dietary interventions and the multi-dimensional nature of cardiometabolic endpoints.

However, recent systematic reviews on GenAI conduct warn that standalone AI systems exhibit non-trivial error rates in data extraction and risk-of-bias appraisal, and face risks regarding reference verification (15, 18). Therefore, the proposed engine relies on an optimized human-AI collaborative paradigm (17). By leveraging Retrieval-Augmented Generation (RAG) to ground LLM outputs in verifiable, curated knowledge bases, the engine mitigates hallucinations and creates a continuously updated “living evidence pool” (11). This engine continuously ingests newly published literature and real-world datasets, translating unstructured medical narratives into machine-actionable knowledge graphs. Consequently, downstream researchers and guideline developers can dynamically extract high-certainty, synthesized evidence on-demand, effectively collapsing the traditional years-long research-to-practice timeline. It is critical to note, however, that while LLM-assisted abstract screening and RAG-based data extraction are current realities, a fully autonomous “living evidence pool” is not yet deployable. The current state-of-the-art remains a semi-automated workflow; achieving a methodologically flawless, completely independent evidence ecosystem is a long-term developmental target rather than an immediate reality.

## Phase 4: public knowledge translation and patient empowerment

The ultimate value of the evidence ecosystem lies in bridging the “last mile” of translational medicine, converting highly structured, evidence-synthesized clinical guidelines into actionable, patient-centric dietary practices. In the management of CMDs, traditional patient education materials frequently exceed average health literacy levels, failing to inspire sustained behavioral modifications. LLMs and conversational AI agents present a transformative opportunity to re-engineer public health communication, serving as 24/7 personal digital nutritionists capable of translating complex clinical heuristics into highly readable, empathetic, and culturally tailored dietary guidance (19, 20).

Recent empirical evaluations underscore the profound capacity of conversational AI to enhance patient support in cardiovascular care. For instance, in addressing inquiries from patients with heart failure regarding fluid restrictions and specialized dietary habits, advanced LLMs have demonstrated exceptional accuracy and reproducibility, rendering clinical definitions in a highly simplified, patient-friendly manner (21). Beyond static informational retrieval, integrated conversational systems possess the potential to revolutionize chronic disease frameworks, such as diabetes and obesity management, by providing real-time patient engagement, personalized meal planning, and continuous emotional or behavioral reinforcement (22). Furthermore, by embedding RAG architectures grounded in authoritative national health databases, next-generation chatbots can deliver interactive, evidence-grounded nutritional explanations that bridge the gap between professional textual ease and experiential clarity for diverse patient populations (23).

However, despite these promising patient-centered capabilities, current foundational models present non-trivial safety risks, occasionally generating misleading statements or demonstrating high variability in scientific completeness when guidelines are absent (24, 25). Computational readability metrics also reveal that standalone AI outputs frequently exceed recommended health literacy benchmarks, presenting a barrier to understandability for the general public (26, 27). To mitigate these hazards, the final phase of our ecosystem advocates for a “clinician-guided AI counseling” paradigm rather than unmonitored patient reliance (28). Within this framework, AI serves as an interactive copilot for healthcare teams, optimizing patient self-management and feedback. Crucially, the real-world behavioral and metabolic tracking data generated during patient adherence are securely fed back into the primary input layers, completing the dynamic loop of the AI-driven evidence ecosystem.

## Translating the ecosystem from concept to implementation

While the conceptual framework provides a strategic blueprint, transitioning this AI-driven ecosystem into clinical reality requires robust technological infrastructure. To bridge the gap between theoretical modeling and operational deployment, we delineate the specific technical pathways for each phase in Table 1. This includes the proposed algorithmic architectures, essential data interoperability standards (e.g., FHIR, OMOP CDM), and the primary technical barriers alongside their corresponding mitigation strategies.

## Discussion and future directions

The conceptualization of an AI-driven evidence ecosystem represents a paradigm shift in cardiometabolic nutrition, yet its realization is heavily contingent upon overcoming substantial ethical, methodological, and infrastructural hurdles. As the ecosystem ingests vast streams of high-dimensional omics data and real-world digital biomarkers, safeguarding patient privacy, ensuring data governance, and mitigating algorithmic biases become paramount. AI models trained on historically skewed or homogenous demographic datasets risk perpetuating health disparities in nutritional recommendations. Consequently, the development of these multi-agent frameworks must strictly adhere to principles of algorithmic transparency and equitable representation.

Beyond ethical considerations, translating this ecosystem into clinical practice faces substantial regulatory, infrastructural, and economic barriers. From a regulatory perspective, AI-driven systems generating individualized nutritional advice must navigate stringent pathways governing Software as a Medical Device (SaMD). Securing requisite approvals, such as FDA clearance or CE marking, is a prerequisite to guarantee clinical safety, accountability, and efficacy. Furthermore, integrating highly heterogeneous data streams, spanning multiomics, wearable continuous monitoring, and clinical trial registries, necessitates the strict adoption of global data interoperability standards, such as HL7 Fast Healthcare Interoperability Resources (FHIR) and the Observational Medical Outcomes Partnership (OMOP) Common Data Model. To secure data privacy during cross-institutional and international collaborations, the ecosystem must employ privacy-preserving technologies such as federated learning, allowing models to train on decentralized multiomics and CGM data without transferring sensitive patient information.

Additionally, deploying and sustaining this ecosystem demands enormous computational resources and human capital. The financial burden of training multi-agent systems, coupled with the ongoing costs of maintaining expert “human-in-the-loop” oversight panels, presents a significant economic challenge. If these infrastructural costs are not carefully managed, there is a profound risk that such advanced precision nutrition tools will be confined to well-funded academic centers, thereby inadvertently widening health disparities in low-resource settings.

Equally critical is the profound impact of this automation on the biomedical workforce. While intelligent evidence synthesis engines drastically reduce cognitive burdens, they simultaneously introduce the risk of “de-skilling” among early-career researchers and clinicians. Over-reliance on AI-generated summaries without rigorous critical appraisal could lead to the uncritical acceptance of algorithmic hallucinations or flawed methodologies. To counteract this, the scientific community must actively redefine medical and methodological education. It is imperative to establish specialized training programs and rigorous methodological workshops focused on advanced techniques, such as AI-enabled network meta-analysis and multi-agent prompt engineering, equipping the next generation of researchers with the hybrid skills necessary to critically navigate and validate these complex systems.

Equally imperative is the establishment of rigorous validation strategies to continuously evaluate the ecosystem’s clinical and methodological utility. Success must be quantified through a multidimensional metric framework. Methodologically, the efficiency of the living synthesis engine can be benchmarked by the reduction in time elapsed from primary trial publication to formal clinical guideline integration. Furthermore, the accuracy of AI agents must be rigorously assessed via non-inferiority study designs, comparing AI-assisted literature screening and data extraction against gold-standard human performance (e.g., Cochrane methodological standards) in terms of sensitivity, specificity, and error rates. Clinically, the ultimate success of the ecosystem relies on measuring quantifiable improvements in patient adherence to precision dietary plans, significant reductions in clinician cognitive burden during routine nutritional counseling, and the statistical correlation between AI-predicted metabolic responses and real-world cardiometabolic outcomes in pragmatic clinical trials.

Ultimately, the proposed four-phase ecosystem, spanning from multiomics decoding and real-world evidence generation to intelligent synthesis and personalized public translation, is not designed to replace human expertise, but rather to augment it. Operating strictly under a “human-in-the-loop” paradigm, AI serves as an indispensable bridge connecting isolated research silos. By transforming fragmented, stagnant data into a dynamic, interconnected, and highly translational evidence network, this intelligent ecosystem holds the promise of fundamentally reshaping the future of precision cardiometabolic care.

## Data Availability

The original contributions presented in this study are included in this article/supplementary material, further inquiries can be directed to the corresponding author.
